# Geographic Disparities of Community-level 65+ Depression Prevalence in Five New England States

**DOI:** 10.1093/geroni/igaf122.2588

**Published:** 2025-12-31

**Authors:** Elizabeth Dugan, Taylor Jansen, Qian Song, Yan-Jhu Su, Shan Qu, Mengshi Liu

**Affiliations:** University of Massachusetts Boston, Boston, Massachusetts, United States; University of Massachusetts Boston, Boston, Massachusetts, United States; University of Massachusetts Boston, Boston, Massachusetts, United States; The Pennsylvania State University, University Park, Pennsylvania, United States; University of Massachusetts Boston, Boston, Massachusetts, United States; University of Massachusetts Boston, Boston, Massachusetts, United States

## Abstract

Rural residents report higher depression rates than their urban counterparts. The present descriptive study will compare community and neighborhood-level 65+ “ever-diagnosed” depression rates in five New England states: Connecticut (CT), Massachusetts (MA), Maine (ME), New Hampshire (NH), and Rhode Island (RI). The Healthy Aging Data Reports (HADRs) (www.healthyagingdatareports.org) are a comprehensive tool to spur community and policy action as they calculate community-level prevalence of 38 chronic diseases for each community in a state. Using Medicare Beneficiary Summary File (2020-2021), representing 100% of traditional beneficiaries. Small area estimation techniques were used to calculate age-sex adjusted community depression rates and over 150 other health indicators. Results varied across states with NH reporting the lowest community rate of 65+ depression in the sample (20.01%) and MA reporting the highest (54.46%). CT and MA reported the largest range in 65+ depression (CT:23.68-42.65%; MA:21.90-54.46%) with the highest rate clusters located near the biggest cities, in urban areas (CT:25.62-42.65%; MA:26.39-54.46%). Conversely, in ME and NH, the highest rate clusters were found in rural areas (NH:29.60-38.08%; ME:36.99-43.40%), states with a more traditional rural/urban threshold compared to the population dense states of CT, MA, and RI. In sum, the 2025 HADRs identified geographic disparities and overall high rate of 65+ community-level depression, with some communities reporting 65+ depression rates higher than 50% (neighborhoods in Springfield & Worcester, MA). The 2025 HADRs provide visualizations to make disparities evident; in this way, they can guide policy efforts to treat and address the growing mental health needs of the aging population.

